# The Importance of Chemokines Activating CXCR1, CXCR2 and CXCR3 in Tumorigenesis as Potential Therapeutic Targets in Monoclonal Gammopathy of Undetermined Significance and Multiple Myeloma

**DOI:** 10.3390/cancers17172888

**Published:** 2025-09-02

**Authors:** Jan Korbecki, Katarzyna Barczak, Beata Bosiacka, Anna Surówka, Ewa Duchnik, Maciej Skarbiński, Emilian Snarski, Dariusz Chlubek, Mateusz Bosiacki

**Affiliations:** 1 Department of Anatomy and Histology, Collegium Medicum, University of Zielona Góra, 28 Zyty St., 65-046 Zielona Góra, Poland; jan.korbecki@onet.eu; 2 Department of Conservative Dentistry and Endodontics, Pomeranian Medical University in Szczecin, 72 Powstańców Wlkp. Av., 70-111 Szczecin, Poland; katarzyna.barczak@pum.edu.pl; 3 Institute of Biology, University of Szczecin, 13 Wąska St., 71-415 Szczecin, Poland; beata.bosiacka@usz.edu.pl; 4 Department of Plastic, Endocrine and General Surgery, Pomeranian Medical University in Szczecin, 72-010 Szczecin, Poland; anna.surowka@pum.edu.pl; 5 Department of the Aesthetic Dermatology, Pomeranian Medical University in Szczecin, 72 Powstańców Wlkp. Av., 70-111 Szczecin, Poland; ewa.duchnik@pum.edu.pl; 6 Institute of Medical Sciences, Collegium Medicum, University of Zielona Góra, 28 Zyty Str., 65-046 Zielona Góra, Poland; m.skarbinski@inm.uz.zgora.pl (M.S.); e.snarski@inm.uz.zgora.pl (E.S.); 7 Department of Biochemistry and Medical Chemistry, Pomeranian Medical University in Szczecin, 72 Powstańców Wlkp. Av., 70-111 Szczecin, Poland; dchlubek@pum.edu.pl

**Keywords:** monoclonal gammopathy of undetermined significance (MGUS), multiple myeloma (MM), CXCL1, IL-8, chemokine, bone marrow, IP-10, MIG, PF4

## Abstract

Multiple myeloma (MM) is a neoplasm of plasma cells. Despite the development of increasingly advanced treatments, multiple myeloma remains challenging to cure completely. Consequently, the underlying mechanisms of this neoplasm are being investigated to identify new therapeutic targets and understand chemoresistance. A particular focus has been placed on the MM bone marrow microenvironment, with chemokines being one of its key components. This review examines the role of chemokines that activate the CXCR2 and CXCR3 receptors in both monoclonal gammopathy of undetermined significance (MGUS) and MM, highlighting all CXC chemokines and their receptors, including CXCL1, CXCL8/IL-8, CXCL9, CXCL10, and platelet factor 4. We focus on the direct effects of selected CXC chemokines on MM cells, specifically their roles in proliferation, migration, interaction with bone marrow cells, the formation of extramedullary disease, and chemoresistance. Additionally, we explore the impact of these chemokines on the MM bone marrow microenvironment, particularly in relation to mesenchymal stromal cells, myeloid-derived suppressor cells, osteoclasts, M2 macrophages, and natural killer cells, as well as processes such as bone destruction and angiogenesis. Finally, we discuss the potential use of drugs targeting the two chemokine axes described, with a focus on inhibitors and adoptive cell therapy.

## Simple Summary

The article examines the role of CXCR1, CXCR2, and CXCR3 ligands in tumor biology in multiple myeloma (MM) and monoclonal gammopathy of undetermined significance (MGUS). Both disorders are characterized by the uncontrolled expansion of plasma cells. The ligands in question are chemokines, which by definition regulate the migration of specific immune cells, but they are also involved in tumor-related processes, including those observed in MM. We describe the contribution of these receptor ligands to proliferation, migration, and chemoresistance of MM cells, and we discuss the therapeutic potential of targeting these proteins in MM.

## 1. Background

Multiple myeloma (MM) is classified under lymphoid neoplasms, specifically plasma cell neoplasms [1]. Each year, approximately 140,000 new cases of MM are diagnosed globally, corresponding to 2.1 cases per 100,000 people [2], and the disease results in nearly 100,000 annual deaths [2]. The median overall survival for MM patients is about five years [3,4]. The highest incidence rates are observed in Australia (5.8 cases per 100,000 people), North America (5.2 cases per 100,000), and Western Europe (4.6 cases per 100,000) [2], and these figures are expected to rise as populations age. Conversely, the lowest incidence rates are found in Africa and Southeast Asia, where diagnoses are about 1 case per 100,000 people [2,5].

MM cells originate during B-cell development in germinal centers [6,7], often resulting from mitotic errors during B-cell proliferation, leading to the formation of hyperdiploid (HY) MM cells, which contain extra chromosomes. This subgroup accounts for about half of MM cases, with the most common trisomies involving chromosomes 3, 5, 7, 9, 11, 15, 19, and 21 [8]. The hyperdiploid subtype is generally associated with a favorable prognosis. Another pathway leading to MM involves erroneous chromosomal translocations during class-switch recombination, where an enhancer from the immunoglobulin heavy locus (*IGH)* gene on chromosome 14 is fused with an oncogene from another chromosome. Common translocations include t(11;14) (oncogene: *CCND1*), t(4;14) (oncogene: *FGFR3*/*MMSET*), t(6;14) (oncogene: *CCND3*), t(14;16) (oncogene: *MAF*), and t(14;20) (oncogene: *MAFB*) [6,7].

*CCND1* and *CCND3* encode cyclin D1 and D3, respectively, whose increased expression accelerates MM cell proliferation, making t(11;14) and t(6;14) translocations result in similar mechanisms in MM cells. MM cells with these translocations fall under the CD1 or CD2 molecular subgroups [9,10], with CD2 distinguished by chromosome 1p gene expression and early B-cell markers such as *CD20*, *VPREB*, and *PAX5*. The MS subgroup, associated with t(4;14), is characterized by elevated expression of *FGFR3* and *MMSET*, linked to a poorer prognosis. FGFR3 is a membrane receptor, while MMSET is a histone methyltransferase, affecting gene expression through chromatin modification. Another adverse prognosis subgroup, MF, is associated with t(14;16) and t(14;20), leading to increased expression of c-MAF or MAFB, respectively, which promotes cyclin D2 expression and MM cell proliferation [10].

Other molecular subgroups include PR, characterized by rapid proliferation and poor prognosis, and LB, marked by low levels of bone destruction and a better prognosis [10].

Following these genomic alterations, B cells migrate to the bone marrow, where they differentiate into plasma cells—a normal physiological process. However, the acquired alterations drive excessive proliferation, eventually leading to monoclonal gammopathy of undetermined significance (MGUS) [6]. MGUS is characterized by elevated M-protein levels in the blood, and its incidence increases with age. By the age of 30–39, initial cases are observed, with prevalence rising to 1.6–3.0% at age 60–69 and 4.2–6.6% in those over 80 [11,12]. About 1.2% of MGUS cases progress to MM or related diseases annually [13]. When plasma cells exceed 10% in the bone marrow and M-protein reaches 30 g/L in the blood, the condition is classified as smoldering MM (sMM) [14], with a 22% chance of progression to MM within two years and a median progression time of 6.4 years [15].

MM compromises bone marrow function and causes bone destruction. Elevated M-protein levels lead to inflammatory kidney damage [16], and MM is characterized by hypercalcemia, renal failure, anemia, and bone lesions (CRAB criteria) [17]. Currently, the SliM CRAB criteria are used in clinical practice, expanding on the traditional CRAB criteria with three additional markers that indicate symptomatic multiple myeloma: **S** (*Sixty*)—≥60% plasma cells in the bone marrow; **Li** (Light chains)—A serum free kappa/lambda light chain ratio >100 or <0.01; **M** (Magnetic Resonance)—More than one lesion characteristic of myeloma detected on MRI These additional criteria enhance the early identification of symptomatic disease [18,19].

The International Staging System (ISS), based on serum β2-microglobulin (B2M) levels, divides MM into three stages, with the revised ISS (R-ISS) also incorporating serum lactate dehydrogenase (LDH) levels and chromosomal abnormalities [20].

A small number of MM cells can escape into the bloodstream [21], with circulating MM cells estimated at 0.9 cells per 1 µL f blood in MM patients. In severe cases, this number increases, and when MM cells exceed 5% of nucleated cells, the condition is classified as plasma cell leukemia [22]. These circulating cells may eventually form tumors outside the bone marrow, leading to extramedullary disease in about 15% of MM patients [23], which is associated with a poorer prognosis [24]. Relapses frequently involve extramedullary sites, occurring in about three-quarters of patients with recurrent MM [23], most commonly in the liver, peritoneal surfaces, and kidneys.

Plasma cell tumors, including extramedullary and marrow plasmacytomas, typically present as a solitary mass either within or outside the bone, most commonly in the upper respiratory tract. They are characterized by normal bone imaging findings (including MRI or CT scans of the spine and pelvis), the absence of CRAB symptoms, and minimal bone marrow involvement, with plasma cells comprising less than 10% of the marrow [25].

MM treatment currently includes seven major drug groups [26,27]. Proteasome inhibitors such as bortezomib and carfilzomib are frequently used alongside immunomodulatory agents such as thalidomide, pomalidomide and lenalidomide, often in combination with steroids such as dexamethasone. Other treatments include alkylating agents (melphalan, cyclophosphamide), anthracyclines (doxorubicin), histone deacetylase inhibitors (panobinostat), and monoclonal antibodies (elotuzumab, daratumumab). Recently, immunotherapy options such as chimeric antigen receptor (CAR) T-cell therapy [28] and T-cell-redirecting bispecific antibodies [29] have been introduced.

Despite the growing arsenal of therapeutic options, MM remains incurable, which underscores the need for continued research into tumor mechanisms to uncover new therapeutic targets and better understand chemoresistance. In the vast majority of patients, multiple myeloma remains incurable; in a small group of patients, allogeneic bone marrow transplantation is considered, which theoretically can provide complete cure.

One promising area of focus is intercellular signaling, particularly involving chemokines, which are chemotactic cytokines [30]. These molecules play a critical role not only in cell migration but also in tumorigenesis. Chemokines are divided into four subfamilies based on conserved N-terminal motifs, with one subfamily being the α-chemokines, which possess a CXC motif at the N-terminus. This group includes 16 members, CXC motif chemokine ligand (CXCL)1 to CXCL17, excluding CXCL15, which is specific to mice. These chemokines activate receptors CXC motif chemokine receptor (CXCR)1-CXCR6.

Currently, no comprehensive review exists summarizing the role of CXC chemokines in MM tumorigenesis. This review aims to fill that gap by focusing on the CXC chemokines that activate CXCR2 and CXCR3 receptors. It seeks to compile all current knowledge on the role of these chemokines in MM progression and assess the potential for targeting these chemokines as a therapeutic strategy.

## 2. CXCR1 and CXCR2

### 2.1. Ligands of Receptors CXCR1 and CXCR2

Chemokines that serve as ligands for CXCR2 (also known as CD182) include CXCL1 (also called Gro-α, MGSA), CXCL2 (Gro-β), CXCL3 (Gro-γ), CXCL5 (ENA-78), CXCL6 (GCP-2), CXCL7 (produced from PPBP through proteolytic cleavage), and CXCL8 (commonly known as interleukin (IL)-8, though it will be referred to as CXCL8 in this review as it focuses on chemokines) [31]. The genes for CXCR2 ligands form a cluster on chromosome 4q12-q13 [32,33], suggesting that they may have originated from the duplication of a single gene. CXCR1 (also called CD181) is activated by CXCL6 and CXCL8, and at much higher concentrations, it is also activated by other CXCR2 ligands [31,34,35]. Together with the CXCR2 pseudogene 1 (CXCR2P1), CXCR1 and CXCR2 form a gene cluster at 2q34-q35, further supporting the idea that these genes arose from a duplication event [36].

CXCR1 and CXCR2 are primarily expressed on neutrophils [30,37], making CXCR2 ligands key chemoattractants for these cells. Both receptors are also present on basophils [30]. CXCR2 is expressed on hematopoietic stem cells, where it plays a crucial role in their self-renewal [38], and it is also found on B cell precursors and memory B cells [30]. Additionally, CXCR2 is expressed on endothelial cells, giving its ligands pro-angiogenic properties [39].

While the role of CXCR2 ligands in solid tumors is well-established, their significance in hematolymphoid malignancies has been relatively underexplored. However, this does not imply that they lack importance in these types of neoplasm.

### 2.2. Expression of CXCR1 and CXCR2 Receptors

MM cells express CXCR1 and CXCR2 receptors [40,41], though this expression is observed in only 10% of patients [42]. Even within individual patients, only about half of the MM cells exhibit these receptors [40], indicating the heterogeneity of MM cells within a single patient. This variability may be linked to the higher expression of CXCR1 and CXCR2 in side population (SP) MM cells [43], which are also marked by elevated CXCL6 expression. SP MM cells are defined as cells that do not stain with Hoechst 33342 dye in contrast to main population (MP) MM cells. SP MM cells may represent cancer stem cells (CSC) [43,44]. This suggests a role for these receptors in the stem-like properties of MM cells.

More than half of patients with circulating MM cells show CXCR1 expression [42], a higher proportion than that observed in bone marrow-resident MM cells. This difference could point to a role for CXCR1 in the migration of MM cells from the bone marrow into the bloodstream. CXCR1 likely plays a significant role in MM tumorigenesis, as its higher expression correlates with a poorer prognosis across MM patients, particularly in those with the HY and MY molecular subgroups. Additionally, there is a trend toward worse prognosis in patients with the CD2 molecular subgroup when CXCR1 expression is elevated [45,46]. This suggests that CXCR1 could be a promising therapeutic target, especially in specific molecular subgroups of MM.

#### 2.2.1. CXCL1 Expression in MM

Serum levels of CXCL1 are elevated in MM patients compared to healthy individuals [47,48], and this increase correlates with the ISS. Moreover, CXCL1 levels in the blood are positively associated with both vascular endothelial growth factor (VEGF) levels and bone marrow microvessel density (MVD) in MM patients, suggesting a connection between CXCL1 and angiogenesis within the bone marrow [47]. However, despite these associations, serum CXCL1 levels do not appear to be significantly linked to the overall prognosis of MM patients [47].

Within the bone marrow, MM cells stimulate mesenchymal stromal cells (MSCs) to produce more CXCL1, a process mediated by the transfer of miR-146a via exosomes from MM cells to MSCs [49]. Interestingly, MM cells themselves express lower levels of CXCL1 compared to normal bone marrow plasma cells [50]. In the MS molecular subgroup of MM, higher CXCL1 expression in MM cells is associated with a trend toward poorer prognosis (*p* = 0.099) [45,46].

#### 2.2.2. CXCL2 Expression in MM

Serum levels of CXCL2 are elevated in MM patients compared to healthy individuals [51], and this increase correlates with B2M levels. Despite this, MM cells themselves may not be the primary source of CXCL2, as its expression is lower in MM cells compared to bone marrow plasma cells [50]. In MM patients with the MS molecular subgroup, higher CXCL2 expression in MM cells is associated with a trend (*p* = 0.10) toward poorer prognosis [45,46].

#### 2.2.3. CXCL3 Expression in MM

CXCL3 has not been extensively studied in MM. Its expression in MM cells is lower than in bone marrow plasma cells [50], but it may play a role in tumorigenesis. Higher CXCL3 expression in MM cells is linked to a worse prognosis in patients with the LB molecular subgroup [45,46]. Similarly, in the MS molecular subgroup, higher CXCL3 expression is associated with a trend (*p* = 0.090) toward worse prognosis [45,46].

#### 2.2.4. CXCL5 Expression in MM

Serum levels of CXCL5 are elevated in MM patients compared to healthy individuals [47,48,52,53], and these levels increase with ISS stages. CXCL5 expression is lower in MM cells than in bone marrow plasma cells [50], but it is produced by osteoclasts, where its expression is higher than in MM cells [54]. CXCL5 may inhibit osteoblast differentiation, contributing to bone destruction in MM patients [53]. Serum CXCL5 levels are not significantly associated with prognosis [47], but in the MY molecular subgroup, higher CXCL5 expression is linked to a poorer prognosis [45,46].

#### 2.2.5. CXCL6 Expression in MM

CXCL6 expression in MM cells is lower than in bone marrow plasma cells [50], but its expression is higher in SP MM cells compared to differentiated MM cells [43]. This suggests its autocrine role in SP MM cells, involving the CXCR1 and CXCR2 receptors. In the MS molecular subgroup, higher CXCL6 expression in MM cells is linked to a worse prognosis [45,46].

#### 2.2.6. CXCL7 Expression in MM

CXCL7 expression is higher in circulating MM cells compared to bone marrow MM cells [55]. In patients with extramedullary disease, CXCL7 levels in the bone marrow are higher than in those without extramedullary disease [56], suggesting a role in MM cell migration from the bone marrow. Interestingly, CXCL7 levels in bone marrow are negatively correlated with blood platelet counts but not with other MM-related markers [56], implying that CXCL7 may not directly contribute to MM development in the bone marrow.

#### 2.2.7. CXCL8 Expression in MM

Serum levels of CXCL8 are higher in MM patients compared to healthy individuals [47,51,52,57,58,59,60,61,62,63]. MGUS patients also have higher CXCL8 levels than healthy individuals [58]. In MM, CXCL8 levels increase with ISS stages [62,63,64,65,66]. While CXCL8 serum levels vary in MM patients, high levels are positively correlated with disease activity markers like B2M [51,63,65], LDH [65], anemia [63], and kidney disease [62,63].

In the bone marrow, CXCL8 levels are elevated in MM patients compared to healthy individuals [58,67], and higher levels are also found in MGUS patients [58]. A higher level of CXCL8 in the bone marrow is due to increased CXCL8 production in bone marrow cells in patients with MM compared to patients with MGUS and healthy individuals [41].

In the bone marrow, CXCL8 is produced both by MM cells and by non-MM cells [68]. Its expression increases when MM cells interact with bone marrow cells. CXCL8 is specifically produced by MM cells [69], and its expression in MM cells is higher than in normal bone marrow plasma cells [68]. The elevated expression of CXCL8 in MM cells is linked to the activation of nuclear factor κB (NF-κB) in these cells, which, among other factors, is driven by the high expression of lncRNA H19 [69]. CD28 also contributes to the increased expression of CXCL8 in MM cells [70,71], which is related to the RE/AP composite element in the *IL8* gene promoter that binds NF-κB and activating protein-1 (AP-1). Additionally, interactions with bone marrow macrophages further enhance CXCL8 production in MM cells [72].

However, MM cells may not be the primary source of CXCL8 in the bone marrow [68]. In more than half of MM patients, CXCL8 is produced by MSCs in the bone marrow microenvironment [64,73], whereas in MGUS patients and healthy individuals, MSCs in the bone marrow produce low levels of CXCL8. MM cells stimulate increased CXCL8 production in MSCs within the bone marrow [49,74], a process linked to the transfer of miR-146a via exosomes from MM cells to MSCs. Additionally, the heightened production of CXCL8 in MSCs in MM patients results from the increased expression of APE1/Ref-1, which also elevates IL-6 production [75]. However, another study did not confirm that MM alters CXCL8 expression in MSCs [76]. Bone marrow endothelial cells also contribute to CXCL8 production in the bone marrow [40].

A lower CXCL8 level in the blood is an indicator of a favorable response to therapy using bortezomib, melphalan and prednisolone (VMP), or bortezomib, melphalan, prednisone and thalidomide (VMPT) [59], or cyclophosphamide, thalidomide and dexamethasone (CTD), or PS-341/bortezomib, adriamycin/doxorubicin, dexamethasone (PAD) [63]. After chemotherapy, serum CXCL8 levels decrease [47,57], while higher blood levels of CXCL8 are associated with a poorer prognosis [59,63,77], indicating that systemic CXCL8 production is linked to the impact of MM.

CXCL8 levels in the blood of MM patients may also predict chemotherapy side effects. Patients with higher serum CXCL8 levels were more likely to experience infections and polyneuropathy after chemotherapy [63,78], whereas lower levels of this chemokine were associated with a higher incidence of lymphopenia [78].

## 3. Bioinformatics Analysis of CXCR1, CXCR2, and Their Ligands in MM Tumorigenesis

Bioinformatics analysis using the GSE4204 dataset [45,46] on the KM-plotter portal (https://kmplot.com/analysis/ accessed on 1 September 2024) revealed that higher CXCR1 expression is linked to worse prognosis in MM patients, particularly in the HY and MY molecular subgroups, with a trend toward poorer prognosis in the CD2 subgroup (Table 1). Different CXCR2 ligands were associated with worse outcomes in specific molecular subgroups, such as CXCL3 in the LB subgroup, CXCL5 in the MY subgroup, and CXCL6 in the MS subgroup. In the MS subgroup, higher expressions of CXCL1, CXCL2, CXCL3, and CXCL8 also trended toward worse prognosis (*p* < 0.10). These findings suggest that the CXCR1:CXCL6/CXCL8 axis plays a key role in MM tumorigenesis, and that different CXCR2 ligands may influence MM progression by acting on non-MM cells in the bone marrow niche. However, these mechanisms remain poorly understood. Interestingly, higher expressions of CXCR2 and its ligands in MM cells is sometimes correlated with better prognosis, hinting at a potential anti-MM role for CXCR2, though this effect may depend on CXCR2 expression in MM cells.

## 4. Involvement of CXCR2 Ligands in Tumorigenesis in MM

CXCR1 and CXCR2 ligands can enhance the proliferation of MM cells, as demonstrated in studies on U266, RPMI-8226, IM9 cell lines, and primary MM cells from patients [40]. This effect may be driven by the action of these ligands on SP MM cells, which express higher levels of CXCR1 and CXCR2 than differentiated MM cells [43]. SP MM cells also show elevated CXCL6 expression, suggesting an autocrine loop involving the CXCL6-CXCR1/2 axis that could explain the association of CXCR1 and CXCL6 expression with poor prognosis in MM patients [45,46].

CXCR2 ligands may also contribute to bone marrow angiogenesis in MM patients. Serum CXCL1 levels positively correlate with serum VEGF levels and MVD in MM patients [47], while CXCL8 production by bone marrow cells is also associated with MVD levels [41]. Additionally, blood levels of CXCL8 correlate with other angiogenic factors, such as angiogenin, platelet-derived growth factor (PDGF)-AB, and VEGF [63,66]. These data suggest that CXCR2 ligands, particularly CXCL1 and CXCL8, are likely involved in the angiogenic processes observed in MM.

CXCR2 ligands are implicated in the worsening of bone destruction in MM. Higher serum CXCL8 levels are associated with MM-related bone disease [62], as these ligands promote osteoclastogenesis and inhibit osteoblast differentiation [53,68]. This may explain why elevated CXCL8 levels correlate with worse prognosis in MM patients [59,63,77].

CXCR2 ligands may also play a role in the development of extramedullary disease. Elevated CXCL7 and CXCL8 levels in the bone marrow plasma of MM patients are associated with extramedullary disease [56,79], suggesting that these ligands may drive MM cells’ egress from the bone marrow. CXCR1 expression is higher in circulating MM cells compared to bone marrow-resident MM cells, indicating that CXCL8, through CXCR1 activation, may contribute to the release of MM cells into the bloodstream [42]. While CXCL8 expression in MM cells is not directly associated with poor prognosis [45,46], higher serum levels of CXCL8 are linked to worse outcomes, likely due to its production by non-MM cells in the bone marrow microenvironment [68]. This could explain the association between higher CXCL8 levels in serum and poorer prognosis [59,63,77].

In addition, CXCL8 is correlated with kidney disease in MM patients [62,63], though the mechanisms behind this remain unclear.

**Figure 1:**
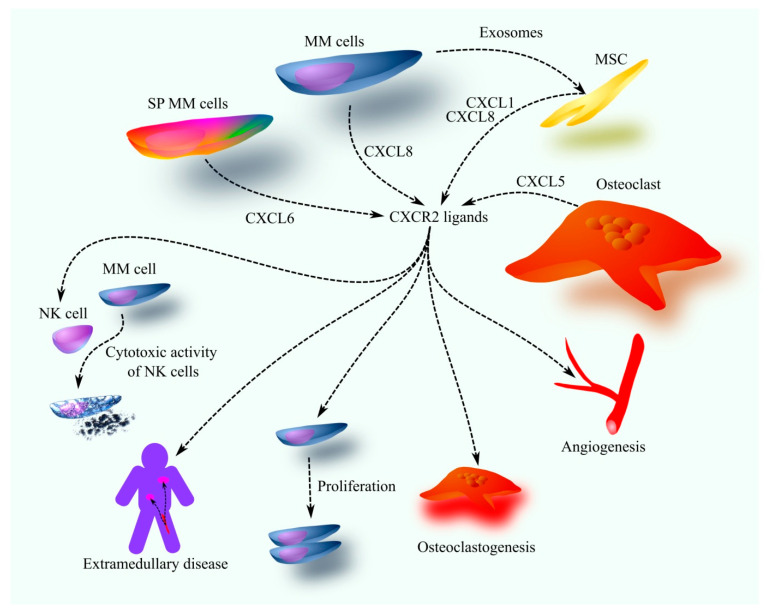
The role of CXCR2 ligands in oncogenic processes in MM. In the MM bone marrow microenvironment, CXCR2 ligands are secreted not only by MM cells but also by other cell types, including osteoclasts and MSC. The production of CXCR2 ligands by MSC is further enhanced by exosomes from MM cells containing miR-146a. Different cell types secrete distinct CXCR2 ligands in significant amounts: MM cells primarily produce CXCL8, SP MM cells secrete higher levels of CXCL6, MSC release CXCL1 and CXCL8, and osteoclasts produce CXCL5. Elevated CXCR2 ligand levels in the MM bone marrow microenvironment drive oncogenic processes through multiple mechanisms. These ligands possess proangiogenic properties, promoting increased bone marrow angiogenesis in MM patients. Additionally, they enhance osteoclast activity, leading to greater bone resorption. CXCR2 ligands also stimulate MM cell proliferation and migration, facilitating their egress from the bone marrow and contributing to the development of extramedullary disease. However, CXCR2 ligands may also exert anti-MM effects by enhancing the cytotoxic activity of NK cells against MM cells.

Interestingly, CXCR2 ligands do not solely promote tumorigenesis in MM (Figure 1). Higher expression of CXCR2 ligands in MM cells is associated with a better prognosis for patients [45,46], possibly due to their role in enhancing NK cell activity. CXCL8, acting through CXCR1 and CXCR2, increases the expression of poliovirus receptor (PVR) ligands on MM cells, which facilitates NK cell recognition and cytotoxic activity against MM cells [80].

### 4.1. CXCR2 Ligands and Therapy Against MM

In vitro studies indicate that CXCL8 may contribute to chemoresistance to velcade (bortezomib) [74]. This suggests that while CXCR2 ligands may play a role in drug resistance, this effect may be specific to certain therapies. Although other studies have shown that CXCL8 does not seem to induce resistance to melphalan, bortezomib, or dexamethasone [68].

CXCL5 and CXCL8 are regulated by NF-κB, making them potential biomarkers for activated NF-κB in MM cells [81]. Drugs that inhibit NF-κB, such as bortezomib, may be particularly effective against MM cells with high CXCR2 ligand expression, potentially explaining the better prognosis in patients with higher expression of these chemokines [45,46].

However, some drugs targeting MM increase CXCL8 production, which may contribute to the promotion of certain neoplasm processes, chemoresistance of MM cells to treatment, and relapse of the disease. Proteasome inhibitors such as bortezomib and epoxomicin increase CXCL8 production in MM cells, even at concentrations lower than those used in therapy [68,82]. This CXCL8 increase is independent of NF-κB and may involve signal transducer and activator of transcription (STAT)3 and STAT6 activation [82]. Other MM drugs, such as lenalidomide and melphalan, also increase CXCL8 expression in MM cells and osteoclasts [68,83]. The impact of these drug-induced increases in CXCL8 on MM tumorigenesis has not been thoroughly studied, but it is clear that CXCL8 has both pro- and anti-tumor properties in MM.

On the one hand, CXCL8 can enhance the vulnerability of MM cells to NK cell attack, potentially improving treatment outcomes [80]. On the other hand, it may promote the proliferation and migration of MM cells [40,42], raising the risk of relapse and treatment failure. Both processes likely occur simultaneously, but the overall effect requires further investigation. Gaining insight into this aspect of anti-MM drugs could lead to the development of more effective drug combinations, aiming to mitigate some of the treatment’s side effects.

The expression of ligands on MM cells is linked to either favorable or unfavorable prognosis, depending on the specific ligand and molecular subgroup of MM. Furthermore, higher expression of CXCR1 and CXCR2 on MM cells is associated with worse and better prognosis, respectively. This suggests that targeting CXCR1 could offer therapeutic benefits for MM patients. However, the effects of CXCR1 inhibitors, CXCR2 inhibitors, and dual CXCR1/2 inhibitors on MM have yet to be explored. The findings from these inhibitors could help clarify how this signaling axis operates in vivo and whether these compounds hold therapeutic potential for MM treatment.

### 4.2. CXCR3

#### 4.2.1. CXCR3 and Its Ligands

The ligands for CXCR3 include CXCL9 (MIG), CXCL10 (IP-10), CXCL11 (I-TAC), and platelet factor 4 (PF4) (CXCL4) [30,31]. The CXCR3 ligand genes form a cluster on chromosome 4q21, while *PF4* resides on chromosome 4q12-q13 along with CXCR2 ligand genes [33]. The CXCR3 receptor, also known as CD183, has three splice variants: CXCR3A, CXCR3B, and CXCR3Alt, each of which responds differently to specific ligands and activates distinct signaling pathways. For instance, CXCL10 does not activate extracellular signal-regulated kinase (ERK) mitogen-activated protein kinase (MAPK) through the CXCR3B variant, unlike other ligands.

CXCR3 is expressed on various immune cells, including NK cells, dendritic cells (DCs), CD4 and CD8 T cells, γδ T cells, and memory B cells [30]. This receptor plays a significant role in immune responses, particularly against tumors [84,85,86]. However, CXCR3 also recruits regulatory T cells (T_reg_) to the tumor microenvironment, inhibiting the immune system’s anti-tumor response [87]. Additionally, CXCR3 can be expressed on endothelial cells during the S/G2-M phase of the cell cycle, where its activation inhibits cell proliferation, giving CXCR3 ligands angiostatic properties [88].

#### 4.2.2. CXCR3 Expression in MM

MM cells express CXCR3 [89,90]. More precisely, MM cells express both CXCR3 isoforms: CXCR3A and CXCR3B, but in varying proportions depending on the cell line and specific MM case [90]. CXCR3 expression on MM cells varies from patient to patient. Typically, about two-thirds of MM cells show CXCR3 expression [40,90]. This variation may be linked to changes in CXCR3 expression during the cell cycle, with higher expression observed in MM cells during the S-M phase [90]. Another factor could be the MM bone marrow microenvironment, where IL-6 and tumor necrosis factor-α (TNF-α) reduce CXCR3 expression in MM cells [90].

CXCR3 receptor expression on MM cells is not present in all patients. Depending on the study, CXCR3 is expressed on MM cells in nearly all patients [91], or in only one-fifth [42]. The level of CXCR3 expression in stage III MM patients is higher than in stage I ISS patients [90]. However, CXCR3 expression levels in MM cells are not correlated with MM cell burden in the bone marrow or the degree of bone destruction caused by MM [90].

#### 4.2.3. PF4 Expression in MM

Serum PF4 levels are lower in MM patients than in healthy individuals, and these levels negatively correlate with B2M and ISS stage [92]. Patients with del(17p) also have reduced serum PF4 levels. A lower PF4 level is linked to a higher likelihood of treatment failure and a worse prognosis, indicating its potential therapeutic role in MM [92]. MM cells show lower PF4 expression than bone marrow plasma cells, a result of *PF4* gene promoter hypermethylation [50].

#### 4.2.4. CXCL9 Expression in MM

Serum levels of CXCL9 are elevated in MM patients compared to those with MGUS and healthy individuals [93]. The levels of all three CXCR3 ligands—CXCL9, CXCL10, and CXCL11—are correlated in the blood of MM patients [93]. Since they activate the same receptor, it is likely that they complement each other in their functions. Serum CXCL9 levels in MM patients are positively correlated with ISS stage, as well as blood levels of LDH and B2M. However, CXCL9 levels are not influenced by cytogenetic aberrations such as del(13q), del(17p), t(11;14), or t(4;14). Higher CXCL9 levels in the blood of MM patients are linked to a worse prognosis [93].

In bone marrow plasma, CXCL9 levels are also higher in MM patients compared to healthy individuals [58,94]. Moreover, MM patients with extramedullary disease exhibit higher CXCL9 levels in the bone marrow compared to those without extramedullary disease [56], indicating a possible mechanism for the release of MM cells from the bone marrow. CXCL9 levels in the bone marrow plasma of MM patients without extramedullary disease increase with ISS stage [56]. However, no significant correlation has been found between bone marrow CXCL9 levels and blood levels of hemoglobin, calcium, creatinine, B2M, or MM burden in these patients [56], suggesting that CXCL9 may not play a central role in tumorigenic mechanisms within the bone marrow.

CXCL9 is expressed in MM cells, similar to other CXCR3 ligands [90,95,96]. Some MM cell lines may lack expression of specific CXCR3 ligands [90,94], implying that CXCR3 ligands could compensate for one another. CXCL9 expression in MM cells is partially dependent on the subtype of MM. In cases with the t(6;14)(p25;q32) translocation, there is overactivation of the multiple myeloma oncogene 1 (MUM1)/interferon regulatory factor 4 (IRF4) [97]. This translocation, which occurs in approximately 1/5 of MM patients, is relatively common [98]. In MM cells with this translocation, MUM1, in cooperation with PU.1, enhances CXCL9 expression [97].

However, it appears that MM cells are not the primary source of CXCL9 in the bone marrow of MM patients [93]. CXCL9 expression in MM cells is lower than in bone marrow plasma cells [93]. Furthermore, certain elements of the MM bone marrow microenvironment, such as IL-6 and TNF-α, can reduce the expression of CXCL9 and other CXCR3 ligands in MM cells [90].

#### 4.2.5. CXCL10 Expression in MM

Serum levels of CXCL10 are higher in MM patients compared to healthy individuals [52,62]. The levels of all three CXCR3 ligands—CXCL9, CXCL10, and CXCL11—are correlated in the blood of MM patients [93]. As the ISS stage progresses, serum CXCL10 levels also increase [62]. Elevated serum levels of CXCL10, along with CXCL9 and CXCL11, are linked to poorer prognosis in MM patients [93], indicating a possible role for CXCR3 ligands in the tumor-promoting processes of MM.

In bone marrow plasma, CXCL10 levels are higher in MM patients compared to healthy individuals [58,94]. Similarly, CXCL10 levels are elevated in the bone marrow plasma of MGUS patients compared to healthy individuals [58]. CXCL10 is expressed in MM cells, similar to other CXCR3 ligands [90,95,96]. Some MM cell lines may lack expression of certain CXCR3 ligands, including CXCL10 [90,94].

The expression level of CXCL10 in MM cells is influenced by the bone marrow microenvironment. Interactions between MM cells and MSCs in the bone marrow lead to increased CXCL10 expression in MM cells [99], and a reciprocal effect occurs, where MM cells stimulate MSCs to produce more CXCL10 [43]. This process is mediated by the transfer of miR-146a via exosomes from MM cells to MSCs. However, within the bone marrow, the expression of CXCL10 and other CXCR3 ligands in MM cells is downregulated by IL-6 and TNF-α [90].

#### 4.2.6. CXCL11 Expression in MM

Serum CXCL11 levels are higher in MM patients than in healthy controls [52], and like CXCL9 and CXCL10, CXCL11 levels are associated with a worse prognosis [93]. CXCL11 levels increase with ISS stage in MM bone marrow [100,101], and higher CXCL11 expression in bone marrow is linked to a worse prognosis [100,101]. CXCL11 expression occurs in MM cells, but some MM lines may lack CXCL11 expression [90,94]. Like other CXCR3 ligands, its expression can be reduced by IL-6 and TNF-α [90], and bone marrow endothelial cells also produce CXCL11 [40].

### 4.3. Bioinformatics Analysis of CXCR3 in MM

Bioinformatics analysis using the GSE4204 dataset [45,46] on the KM-plotter portal (https://kmplot.com/analysis/ accessed on 1 September 2024) showed that CXCR3 expression in MM cells is not generally associated with prognosis, although higher expression in the CD1 molecular subgroup correlates with better prognosis (Table 2). There is also a trend toward better prognosis in PR and MY molecular subgroups, while in the MS molecular subgroup, higher CXCR3 expression is linked to worse prognosis. This suggests that CXCR3 activation may not be a key player in MM tumorigenesis, although its role in non-MM cells may still be important.

CXCL9, in particular, shows a complex relationship with prognosis depending on the molecular subgroup of MM. Higher CXCL9 expression is associated with a worse prognosis in the HY, MF, and MY subgroups, but with a better prognosis in the PR and MS subgroups, indicating its dual role in MM progression.

## 5. Involvement of CXCR3 Ligands in Tumorigenesis in MM: An MM-Enhancing Effect

CXCR3 ligands may promote MM development. CXCL10 can induce the recruitment of γδ T cells to the MM bone marrow microenvironment [99], which is characterized by hypoxic conditions. These conditions increase the expression of steroid receptor coactivator 3 (SRC-3) in γδ T cells, leading to elevated IL-17 production [99]. IL-17 promotes MM development in the bone marrow [102], suppresses immune responses [102], and enhances osteoclast formation [103].

CXCR3 ligands also affect NK cells by reducing their homing to the bone marrow [104], which is associated with a decreased ability to migrate to CXCL12. However, the number of NK cells in the bone marrow of MM patients does not differ significantly from that in MGUS patients [105], suggesting this mechanism may only occur in vivo. MM patients with higher blood levels of CXCL10 have more CD56^high^CD16^+/−^ NK cells in the bone marrow [105]. Additionally, in patients with lower blood levels of CXCL10, the expression of NKp30 in CD56^low^CD16^high^ NK cells is higher than in MGUS patients, while this relationship is absent in patients with high CXCL10 levels. Higher expression of NKp30 in MM patients is associated with better prognosis, as it indicates antitumor activity of NK cells [106]. This suggests that in patients with low levels of CXCL10 in the blood, NK cells fight MM more effectively [105,106].

CXCL11 produced by MM cells increases the number of M2 macrophages in the bone marrow microenvironment [101], promoting macrophage polarization toward an immunosuppressive M2 phenotype that supports tumor growth [107]. CXCR3 ligands also act directly on MM cells. CXCR3 can serve as a protective mechanism against FAS-induced apoptosis. During FAS activation, MM cells increase the production and secretion of CXCL10 and upregulate CXCR3 expression on their cell membranes. Activation of CXCR3A has an anti-apoptotic effect, inhibiting FAS-mediated apoptosis [90].

**Figure 2:**
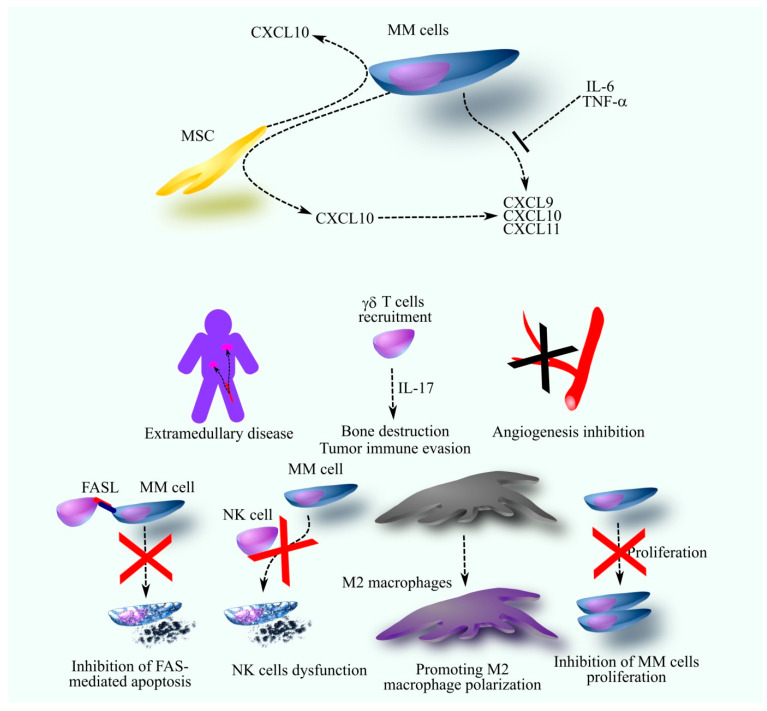
The role of CXCR3 ligands in oncogenic processes in MM. In the MM bone marrow microenvironment, MM cells produce CXCR3 ligands, including CXCL9, CXCL10, and CXCL11. The production of these ligands by MM cells is downregulated by cytokines such as IL-6 and TNF-α. However, at least CXCL10 production is enhanced through interactions between MM cells and MSC. MSC also produce CXCL10, and their interaction with MM cells further increases its secretion. CXCR3 ligands play a dual role in the MM bone marrow microenvironment, exhibiting both pro- and anti-MM effects. Their anti-MM properties include inhibiting angiogenesis and suppressing MM cell proliferation. Conversely, they can also promote MM progression by inducing M2 macrophage polarization, impairing NK cell function, and inhibiting FAS-mediated apoptosis in MM cells. Furthermore, CXCR3 ligands recruit γδ T cells to the bone marrow, which secrete IL-17—a cytokine that enhances bone destruction and facilitates tumor immune evasion. CXCR3 ligands are also implicated in the development of extramedullary disease.

Higher CXCL9 levels in the bone marrow of MM patients with extramedullary disease suggest a mechanism for MM cell release from the bone marrow (Figure 2) [56]. CXCR3 activation induces chemotaxis and upregulates matrix metalloproteinase (MMP)2 and MMP9 expression [91], which may lead to MM cell egress from the bone marrow.

## 6. Involvement of CXCR3 Ligands in Tumorigenesis in MM: An Anti-MM Effect

CXCR3 ligands can exhibit anti-MM effects in some tumor processes, such as angiostasis. They inhibit endothelial cell migration [108] and proliferation [109], potentially suppressing angiogenesis in the bone marrow of MM patients. CXCR3 activation by CXCL10 can reduce MM cell proliferation [109], and CXCR3 ligands can counteract IL-6-induced MM cell proliferation [90]. However, other studies suggest CXCR3 ligands do not affect MM cell proliferation [91], while some research shows CXCL11 can increase MM cell proliferation in an autocrine manner [101].

Despite some anti-MM properties, the pro-MM effects of CXCR3 receptor ligands appear to be stronger [45,46].

### 6.1. Anti-MM Properties for PF4

The anti-MM properties of PF4 are attributed to its second receptor, LRP1 [110]. PF4 reduces IL-6-induced STAT3 activation in MM cells, leading to MM cell apoptosis and reduced tumor growth in the bone marrow. PF4 also decreases VEGF production in MM cells and directly inhibits MM-induced angiogenesis in the bone marrow by acting on endothelial cells [110,111]. In vivo studies show that intravenous administration of PF4 increases survival in mice with MM [110].

### 6.2. CXCR3 Ligands and Therapy Against MM

In vivo studies in MM mouse models show that intravenous PF4 administration increases survival, independent of CXCR3 [110]. Higher PF4 expression in MM cells is associated with better prognosis in MM patients [45,46], suggesting that PF4 administration may have therapeutic potential. However, in the PR molecular subgroup of MM, higher PF4 expression is associated with a trend toward worse prognosis (*p* = 0.053) [45,46], indicating that PF4 therapy may be effective only in specific MM molecular subgroups. Further investigation into this relationship and its mechanisms is needed.

In vivo studies show that CXCL10 overexpression in MM cells inhibits MM development [109], suggesting that either increasing CXCL10 expression in the bone marrow or administering CXCR3 ligands could improve patient outcomes. However, these effects may be limited to animal models. In MM patients, higher levels of CXCL11 in serum and bone marrow plasma CXCR3 ligands are associated with worse prognosis [93,100,101]. Given the many pro-MM properties of CXCR3 ligands, their administration in MM patients could worsen the disease.

CXCR3 ligands are pro-inflammatory chemokines that can enhance the efficacy of adoptive cell therapy. For example, higher serum CXCL10 levels are associated with better outcomes in B-cell maturation antigen (BCMA)-targeted CAR T cell therapy [112]. CXCL10 increases the proliferation and cytotoxic capacity of BCMA-targeted CAR T cells and decreases programmed cell death-1 (PD-1) expression on these cells, protecting them from exhaustion caused by programmed cell death ligand 1 (PD-L1) activation [112].

In adoptive cell therapy, activated NK cells are also used, but one challenge in MM therapy is that the disease is localized in the bone marrow. Lymphocytes used in such therapy must be able to infiltrate the bone marrow. NK cells activated by IL-15 alone show low CXCR4 expression [113], leading to poor bone marrow infiltration. Reducing CXCR3 expression or activity could improve NK cell homing to the bone marrow, as CXCR3 activation reduces migration to CXCL12 [104]. Alternatively, using NK cells activated with IL-12, IL-15, and IL-18 can improve their bone marrow infiltration ability [113].

## 7. Conclusions

In this review we described the significance of CXCR1, CXCR2 and CXCR3 receptors and their ligands in tumor processes in MM and MGUS. The proteins discussed have been well studied in these processes and are also well understood in relation to clinical parameters in MM and MGUS patients.

Nevertheless, there are still certain gaps in knowledge about these proteins in MM. Higher expression of CXCR2 in MM cells, and in most cases of its ligands, is associated with better prognosis in MM patients. In contrast, expression of the receptor for CXCL8/IL-8 and CXCL6, namely CXCR1, is associated with worse prognosis. Thus, the activity of CXCR1/CXCR2 ligands in the MM bone marrow microenvironment is antitumor in MM, while activation of CXCR1 on MM cells is protumor, unlike activation of CXCR2 on MM cells.

The exact significance of CXCR1 in MM cells is not yet known. However, the indicated relationships suggest that this receptor may be an interesting therapeutic target in MM therapy. For this reason, the role of CXCR1 in MM cells in tumor processes should be investigated in the future. The potential of therapy targeting CXCR1 in MM treatment should also be evaluated.

Another topic discussed in this review is the role of the CXCR3 axis in MM tumor processes. Higher expression of CXCL9 in MM cells, as well as higher levels of CXCR3 ligands in MM patients, is associated with worse prognosis. Many studies cited in this review show the involvement of CXCR3 ligands in protumor processes in MM. Therefore, the next direction of research should be the development of therapy targeting the CXCL9–CXCR3 axis, as well as incorporating such therapy into standard MM treatment.

## Figures and Tables

**Table 1:** Association of CXCR1 and CXCR2 receptor expression levels along with ligands on survival of patients with MM relative to the molecular subgroup of MM [45,46].

Gene	In All Patients	CD1	CD2	PR	HY	LB	MF	MS	MY
*CXCR1*	↓	-	↓ *p* = 0.055	-	↓	-	-	-	↓
*CXCR2*	↑	↑ *p* = 0.051	-	-	↑ *p* = 0.054	-	-	-	-
*CXCL1*	↑	-	-	↑	↑	-	-	↓ *p* = 0.099	-
*CXCL2*	↑	-	-	-	-	-	-	↓ *p* = 0.10	↑ *p* = 0.096
*CXCL3*	-	-	-	-	↑	↓	-	↓ *p* = 0.090	-
*CXCL5*	↑	-	-	↑	-	-	-	-	↓
*CXCL6*	-	↑ *p* = 0.10	-	-	-	-	-	↓	-
*PPBP*	↑	-	-	-	↑	-	-	-	↑ *p* = 0.10
*CXCL8*	↑	↑	-	-	-	-	-	↓ *p* = 0.086	-

↑ blue background—higher expression is associated with better prognosis. ↓; red background—higher expression is associated with worse prognosis.

**Table 2:** Association of CXCR3 receptor expression levels along with ligands on survival of patients with MM relative to the molecular subgroup of MM [45,46].

Gene	In All Patients	CD1	CD2	PR	HY	LB	MF	MS	MY
*CXCR3*	-	↑	-	↑ *p* = 0.056	-	-	-	↓ *p* = 0.071	↑ *p* = 0.094
*PF4*	↑	-	-	↓ *p* = 0.053	↑	-	-	-	-
*CXCL9*	↓	-	-	↑	↓	-	↓	↑	↓
*CXCL10*	-	-	-	↑	-	-	-	-	↓
*CXCL11*	-	-	-	↓ *p* = 0.10	-	-	-	-	↑ *p* = 0.099

↑ blue background—higher expression is associated with better prognosis. ↓; red background—higher expression is associated with worse prognosis.

## Data Availability

Data is provided within the manuscript.

## List of Abbreviations

AP-1—activating protein-1; B2M—β2-microglobulin; BCMA—B-cell maturation antigen; CAR—chimeric antigen receptor; CRAB—hypercalcemia, renal failure, anemia, and bone lesions; CXCL—CXC motif chemokine ligand; CXCR—CXC motif chemokine receptor; CXCR2P1—CXCR2 pseudogene 1; DCs—dendritic cells; ERK—extracellular signal-regulated kinase; IGH—immunoglobulin heavy locus; IL—interleukin; IRF4—interferon regulatory factor 4; ISS—international staging system; LDH—lactate dehydrogenase; MAPK—mitogen-activated protein kinase; MDSC—myeloid-derived suppressor cells; MM—multiple myeloma; MMP—matrix metalloproteinase; MSC—mesenchymal stromal cells; MUM1—multiple myeloma oncogene 1; MVD—microvessel density; NF-κB—nuclear factor κB; NK—natural killer; PD-1—programmed cell death-1; PDGF—platelet-derived growth factor; PD-L1—programmed cell death ligand 1; PF4—platelet factor 4; PVR—poliovirus receptor; sMM—smoldering multiple myeloma; SP—side population; SRC-3—steroid receptor coactivator 3; STAT—signal transducer and activator of transcription; TNF-α—tumor necrosis factor-α; Treg—regulatory T cells; VEGF—vascular endothelial growth factor.
